# Real-World Experience of Midodrine in Hospital Setting in Pulmonary Arterial Hypertension

**DOI:** 10.1016/j.chpulm.2025.100214

**Published:** 2025-09-30

**Authors:** Noah Giese, Zeenat Safdar, Anisha Mulumudi, Sarah Momin, Nelson Villasmil Hernandez, Duc T. Nguyen, Edward A. Graviss

**Affiliations:** a Internal Medicine, Houston Methodist Hospital, Houston, TX; b Houston Methodist Lung Center, Division of Pulmonary, Critical Care & Sleep Medicine, Houston Methodist Hospital, Houston, TX; c Pathology and Genomic Medicine, Houston Methodist Research Institute, Houston, TX

**Keywords:** adrenergic agonist, B-type natriuretic peptide (BNP), bradycardia, endothelial receptor antagonists, epoprostenol, hypotension, mean arterial pressure (MAP), midodrine, phosphodiesterase inhibitors, prostaglandins, pulmonary arterial hypertension (PAH), pulmonary vascular resistance, selexipag, systemic vascular resistance, treprostinil, right-sided heart failure

## Abstract

**Background:**

Pulmonary arterial hypertension (PAH), a progressive disease, is characterized by increased pulmonary vascular resistance (PVR) and leads to right ventricular failure and premature death. PAH therapies aim to reduce PVR; however, these treatments as vasodilators may also result in reduced systemic vascular resistance and mean arterial pressure (MAP), leading to clinical or symptomatic hypotension. Low MAP can limit the administration of optimal dosage of PAH drugs. Midodrine, an oral alpha-1 adrenergic agonist, as a promising intervention, can potentially increase mean MAP and improve tolerance to PAH therapies.

**Research Question:**

Does the use of midodrine improve MAP and allow for simultaneous uptitration of PAH therapy while hospitalized?

**Study Design and Methods:**

A retrospective analysis of 433 patients treated at Houston Methodist Lung Center was undertaken between January 2005 and September 2022. Of these, 57 patients were prescribed midodrine during their hospital stay. We matched 57 patients 1:1 with propensity score matching between patients with PAH not given midodrine (control patients) based on age, sex, World Health Organization functional class, B-type natriuretic peptide, and 6-minute walk distance.

**Results:**

Among hospitalized patients with PAH, those receiving midodrine were more likely to undergo uptitration of their PAH medications compared with those not receiving midodrine (n = 30 vs n = 17, respectively; *P* < .05). Patients on midodrine during hospitalization received higher doses of epoprostenol (*P* < .001), treprostinil (*P* < .05), and selexipag (*P* < .05). Additionally, no adverse effects attributable to midodrine were reported.

**Interpretation:**

This study, to our knowledge the first large-scale analysis of PAH data, investigated the use of midodrine in hospitalized patients with PAH. In this single-center study, we share real-world experience of using midodrine to mitigate systemic hypotension, thereby facilitating the uptitration of PAH-targeted therapies.

**Take-Home Points**

**Study Question:** Does the use of midodrine improve mean arterial pressure and allow for simultaneous uptitration of pulmonary arterial hypertension (PAH) therapy in hospitalized patients?

**Results:** Patients exposed to midodrine underwent uptitration of their PAH medications, including epoprostenol, treprostinil, and selexipag during admission, and experienced improvement in mean arterial pressure with no reported side effects.

**Interpretation:** Our results suggest that midodrine can be used as adjunctive therapy for patients with PAH with treatment-related hypotension and allows for further uptitration of PAH medications.

Pulmonary arterial hypertension (PAH) is a progressive disease that leads to right-sided heart failure and premature death.^1^ Over the past several decades, multiple therapies have been approved for PAH treatment. Because PAH therapies are primarily vasodilators, their effects extend beyond the reduction in pulmonary vascular resistance (PVR), and reduction in mean BP (mean arterial pressure [MAP]) is commonly encountered.^2^, ^3^, ^4^, ^5^, ^6^ This represents a significant challenge for clinicians because patients with PAH require uptitration of medications to clinically optimal doses. Suboptimal dosing of parenteral prostacyclin has been associated with poor outcomes.^3^^,^^6^^,^^7^ Midodrine, an oral alpha-1 adrenergic agonist, has been used to mitigate the reduction in MAP.^7^, ^8^, ^9^, ^10^, ^11^, ^12^

Midodrine exerts its effects through the vasoconstriction of arterioles and the venous capacitance vessels, and these play an important role in regulating systemic venous and arteriolar resistance. It is widely used to augment MAP in orthostatic hypotension and in patients undergoing hemodialysis.^8^, ^9^, ^10^, ^11^, ^12^, ^13^ Previously, a small study suggested a role of midodrine in elevating MAP and improving medication tolerance among patients with PAH.^14^^,^^15^ Although the use of midodrine in PAH has increased, its safety and utilization in a large PAH cohort has not been reported. In this single-center study, we share real-world experience of using midodrine in a hospital setting to mitigate systemic hypotension, thereby facilitating the uptitration of PAH-targeted therapies.

## Study Design and Methods

### Study Design

**Figure 1:**
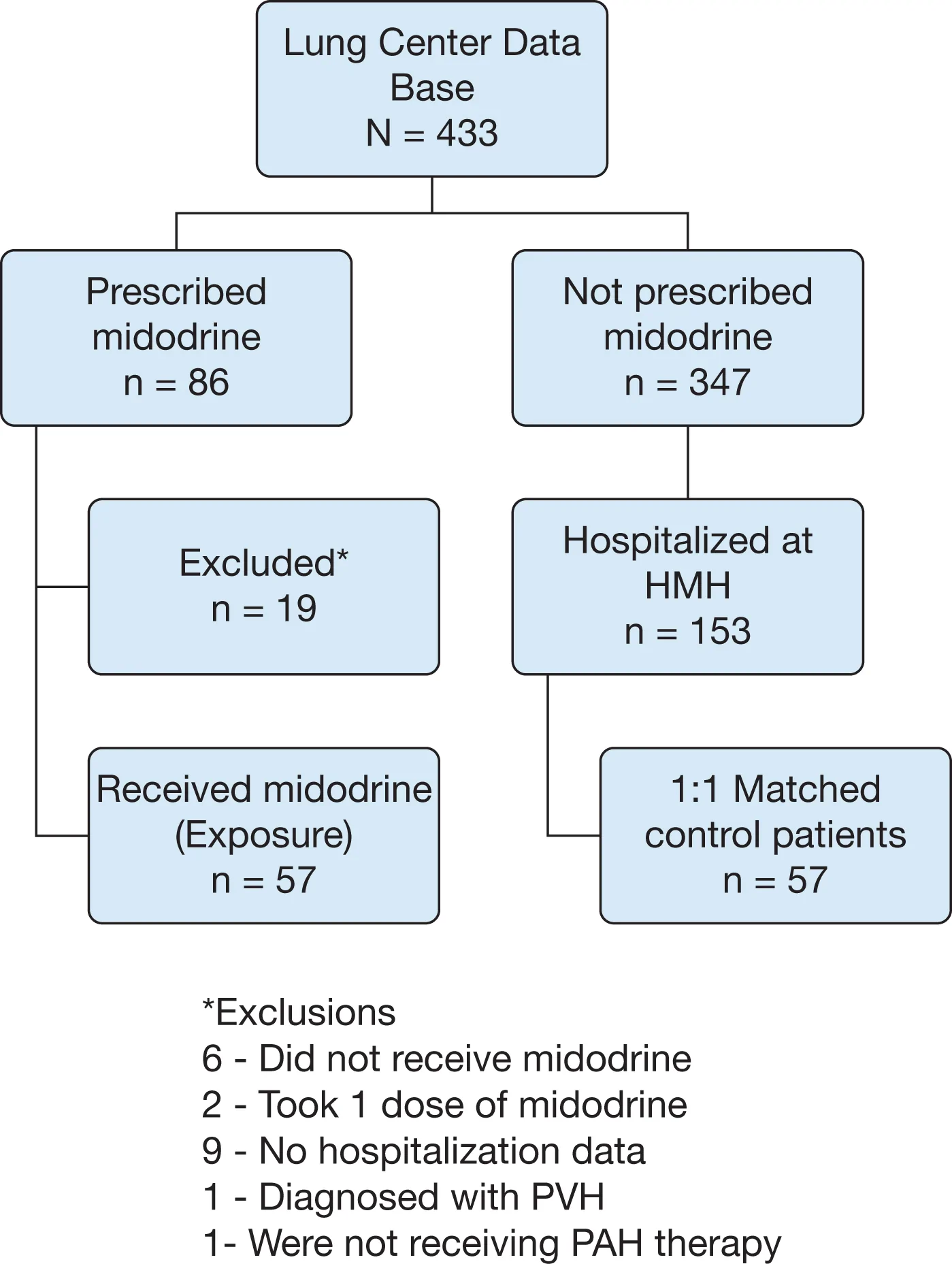
Schema of patients included in the study. HMH = Houston Methodist Hospital; PAH = pulmonary arterial hypertension; PVH = pulmonary venous hypertension.

A retrospective analysis of the PAH registry at the Houston Methodist Lung Center, Houston, Texas, was conducted. This work was approved by the Houston Methodist Research Institute institutional review board (No. MOD00007239). All patients included in the study had hemodynamically confirmed PAH, defined as mean pulmonary artery pressure > 25 mm Hg, a pulmonary capillary wedge pressure ≤ 15 mm Hg, and PVR > 3 Wood units. Each patient’s chart was searched for the terms midodrine and ProAmatine between April 7, 2016, and August 1, 2022, with the final vital status determined on March 31, 2023. A total of 86 patients were prescribed midodrine, of whom 19 were excluded (Fig 1). The remaining 57 patients were matched 1:1 with propensity score matching with a control group of patients with PAH who did not receive midodrine. A total of 347 patients with PAH who were not prescribed midodrine and had a hospitalization event between April 7, 2016, and August 1, 2022, were used as control patients.

Using a standard protocol, BP data were collected thrice daily, approximately 4 hours apart, to minimize sampling bias and data sampling errors. In the midodrine group, BP was recorded 4, 8, and 12 hours before midodrine was administered (predose). After administration of 2 consecutive doses of midodrine approximately 8 hours apart, BP was recorded at 12, 16, and 20 hours (postdose). Finally, BP was recorded at 4, 8, and 12 hours before discharge (discharge dose). Because this was a retrospective study, the BP threshold for midodrine administration was at the discretion of the provider. The data collection strategy for the control group was similar, except predose and postdose were calculated before and after being started on home PAH medications, respectively. Baseline right heart catheterization data up to 2 years before admission were obtained. Hospitalizations and transplant data were collected. Data for each patient were systematically recorded in a secure REDCap database, and EPIC electronic medical records values were uploaded into the Houston Methodist Lung Center REDCap database.

### Data Analysis

Propensity score matching (1:1, calibration 1) was performed between patients who received midodrine (midodrine group) and those who did not receive midodrine (control group). Matching criteria included age, sex, race/ethnicity, BMI, estimated glomerular filtration rate, duration of disease, brain natriuretic peptide, and world health organization functional class. Sexwas chosen to help control for female predominance of PAH.^16^ Demographic and clinical data were reported as median and interquartile range (IQR) for continuous variables and as frequencies and proportions for categorical variables. Differences between midodrine and control groups were compared using the Wilcoxon signed rank test for continuous variables and McNemar χ^2^ for categorical variables. MAP and average heart rates at predose, postdose, and discharge dose were visualized using box plots, and differences between time points were compared using the Wilcoxon rank sum test. Model fitness was checked with pseudo-*R*^2^. Collinearity was checked with the variance inflation factor. Changes in dosage were calculated as absolute change and absolute mean change in dosage, indicating the difference between the admission and discharge dosages.^17^ This is because when patients start a new medication, they typically initiate the medication at a low dose that is gradually escalated. For example, if 9 patients are each using a medication at 10 mg/d and undergo no change in their dosage, when a 10th patient starts the medication at 5 mg/d, the average dose goes down from 10 mg/d to 9.5 mg/d. This action does not accurately represent what really happened. The change in absolute average dose for this group is 0.5 mg/d, which accurately depicts the dose. For this reason, these data were not presented as median or mean. Analyses were performed using Stata version 17.0 (StataCorp LLC), with statistical significance set at *P* < .05.

## Results

### Baseline Demographics

Demographic data are outlined in Table 1. The etiology of PAH was mainly idiopathic or associated with connective tissue disease. However, a higher number of patients with portopulmonary hypertension were observed in the midodrine group (11 vs 2 patients; *P* = .01). The midodrine group had a shorter 6-minute walk distance (*P* = .046), and a higher REVEAL Lite 2 score (*P* = .03) and B-type natriuretic peptide levels (*P* = .04). This suggests that at baseline, patients that received midodrine likely had more severe disease.

**Table 1:** Baseline Characteristics

Characteristic	Total (n = 114)	Control Group (n = 57)	Midodrine Group (n = 57)	Standardized Mean Difference	*P* Value
Age, y, median	53	51	57	18.8	.32
Vital status				56.0	.68
Alive	80	41	39		
Deceased	34	16	18		
Sex				–13.5	.47
Male	21	9	12		
Female	93	48	45		
Race/ethnicity					
White	49	26	23	–10.6	.57
Black	19	9	10	4.7	.80
Hispanic	37	17	20	11.2	.55
Asian	5	3	2	–8.5	.65
Not specified	4	2	2	0.0	1.00
Etiology of PAH					
Idiopathic pulmonary hypertension	40	22	19	3.8	.83
Hereditable	4	2	2	0.0	1.00
Drug and toxin-induced PAH	5	2	3	7.2	.70
PAH associated with					
Connective tissue disease	41	24	17	–17.7	.35
Congenital heart disease	7	3	4	–13.6	.47
HIV	3	1	2	10.9	.56
Portopulmonary hypertension	13	2	11	59.0	.002
WHO functional class					
I	1	0	1	19.2	.38
II	15	9	6	–27.9	.17
III	53	24	29	–6.8	.74
IV	16	8	8	–11.2	.59
Not assessed/unknown	29	16	13	54.2	.01
6-min walk distance, m	320 (246-419)	360 (314-447)	300 (217-365)	–60.4	.004
REVEAL Lite 2 score	7.0 (6.0-9.0)	7.0 (6.0-8.0)	8.0 (6.0-9.0)	63.3	.11
B-type natriuretic peptide, pg/mL	165 (67-344)	134 (54-287)	192 (72-438)	6.3	.81

Data are presented as No., median (interquartile range), or as otherwise indicated. Differences between midodrine and control groups were compared by the Wilcoxon sign rank test for continuous variable and by McNemar χ^2^ for categorical variables. PAH = pulmonary arterial hypertension; WHO = World Health Organization.

### Baseline Hemodynamics

Baseline echocardiogram data showed increased right atrial area, right atrial index, and right atrial volume, and lower stroke volume in the midodrine group (e-Table 1). Baseline invasive hemodynamics were similar between the 2 groups (Table 2) except for lower stroke volume in the midodrine group (*P* = .02).

**Table 2:** Baseline Right Heart Catheterization

Variables	Total	Control	Midodrine	*P* Value
	(n = 114)	(n = 57)	(n = 57)	
RA mean, mm Hg	8.0 (6.0-14.0)	7.5 (6.0-12.0)	8.0 (6.0-15.0)	.27
PA mean, mm Hg	50.0 (43.0-57.0)	50.0 (47.0-57.0)	49.0 (43.0-56.0)	.38
PCWP, mm Hg	11.0 (8.0-15.0)	9.0 (7.5-14.5)	12.0 (8.0-17.0)	.12
TD CO, L/min	4.7 (3.7-6.0)	5.2 (4.2-6.2)	4.5 (3.5-5.7)	.13
TD CI, L/min/m^2^	2.6 (2.1-3.4)	3.1 (2.4-3.4)	2.5 (2.0-3.2)	.15
HR, beats/min	79.5 (73.0-88.0)	77.0 (74.0-85.0)	82.0 (73.0-91.0)	.49
PVR, WU	8.0 (5.4-10.9)	8.4 (6.3-11.0)	7.4 (5.1-10.9)	.63
TPG, mm Hg	39.0 (28.0-44.0)	40.0 (33.5-46.0)	35.0 (25.0-43.0)	.18
DPG, mm Hg	21.0 (15.0-28.0)	23.0 (15.0-32.0)	20.0 (12.0-26.0)	.14
SV, mL	59.2 (46.2-78.7)	72.0 (58.3-82.2)	51.2 (39.0-67.1)	.02
Pulmonary compliance, mL/mm Hg	1.1 (0.9-1.9)	1.6 (1.1-2.1)	1.1 (0.8-1.5)	.14
Arterial saturation %	95.0 (93.0-98.0)	94.5 (92.4-98.0)	95.3 (93.0-98.0)	.48
MVO_2_, %	67.8 (60.1-72.8)	69.1 (59.3-72.0)	67.0 (60.3-73.7)	.94

Data are presented as median (interquartile range) or as otherwise indicated. Differences between midodrine and control groups were compared by the Wilcoxon sign rank test for continuous variable and by McNemar χ^2^ for categorical variables. DPG = diastolic pulmonary gradient; HR = heart rate; MVO_2_ = mixed venous oxygen saturation; PA = pulmonary artery; PCWP = pulmonary capillary edge pressure; PVR = pulmonary vascular resistance; RA = right atrium; SV = stroke volume; TD CI = cardiac index thermodilution; TD CO = thermodilution cardiac output; TPG = transpulmonary gradient; WU = Wood units.

### Baseline PAH Medications

The PAH medications are outlined in e-Tables 2 and 3, and the summary of medication dose changes are outlined in e-Table 4. Given the severity of disease, 96% of all patients were on dual or triple therapy. The combination of prostacyclin analogs and endothelin receptor antagonist (ERA) was more commonly prescribed in the midodrine group (*P* < .001), whereas phosphodiesterase 5 inhibitors (PDE5-is) were prescribed more in the control group (*P* = .001). The dosages of PAH therapy before admission were similar between the control group and midodrine groups using the Mann-Whitney *U* test. The ERAs were prescribed for 86% and 93% and prostacyclin analogs were prescribed for 77% and 91% of patients in the control group and midodrine groups, respectively.

### BP and Heart Rate Measurements

**Figure 2:**
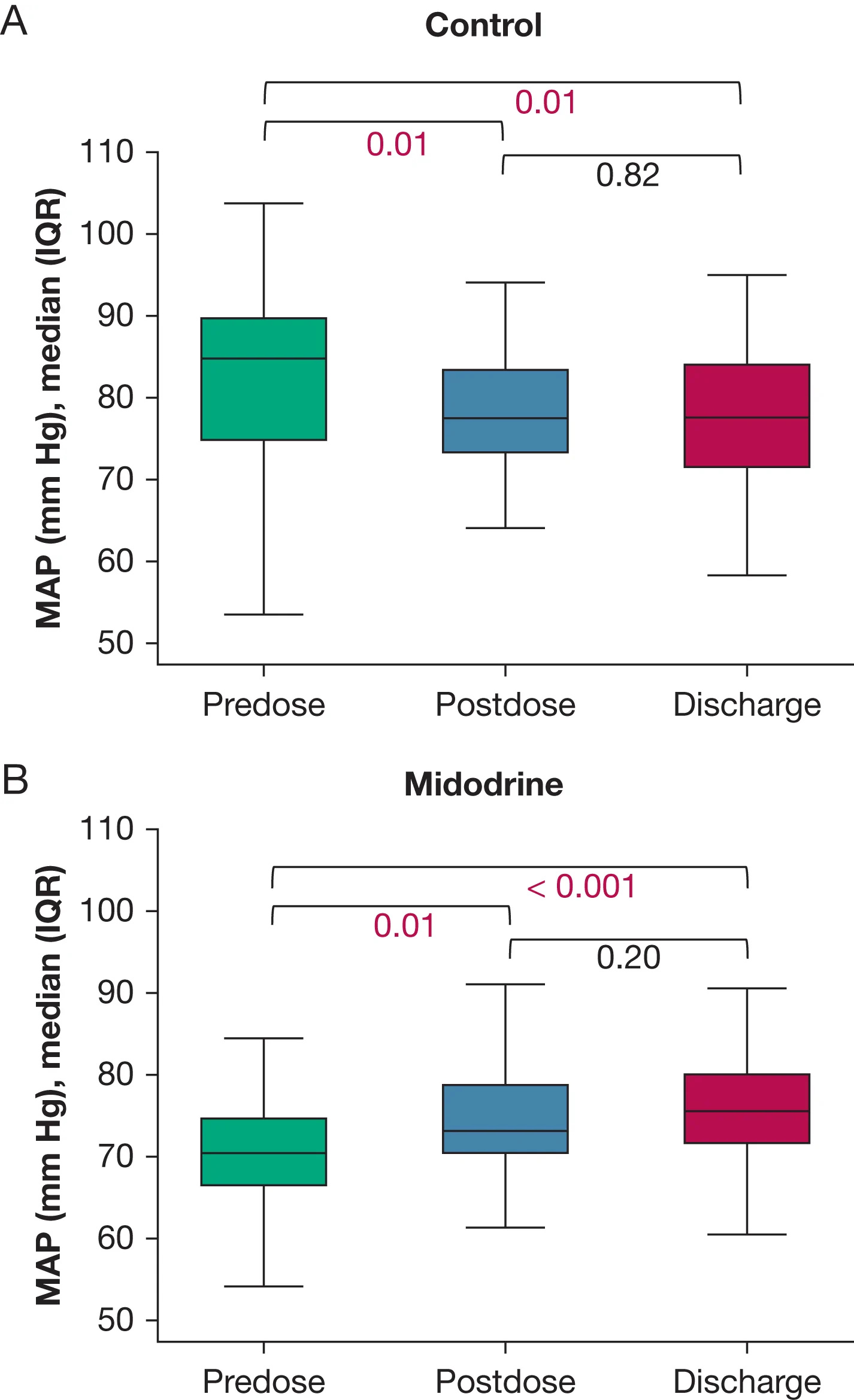
A, B, Mean arterial pressure at 3 time points. IQR = interquartile range; MAP = mean arterial pressure.

**Figure 3:**
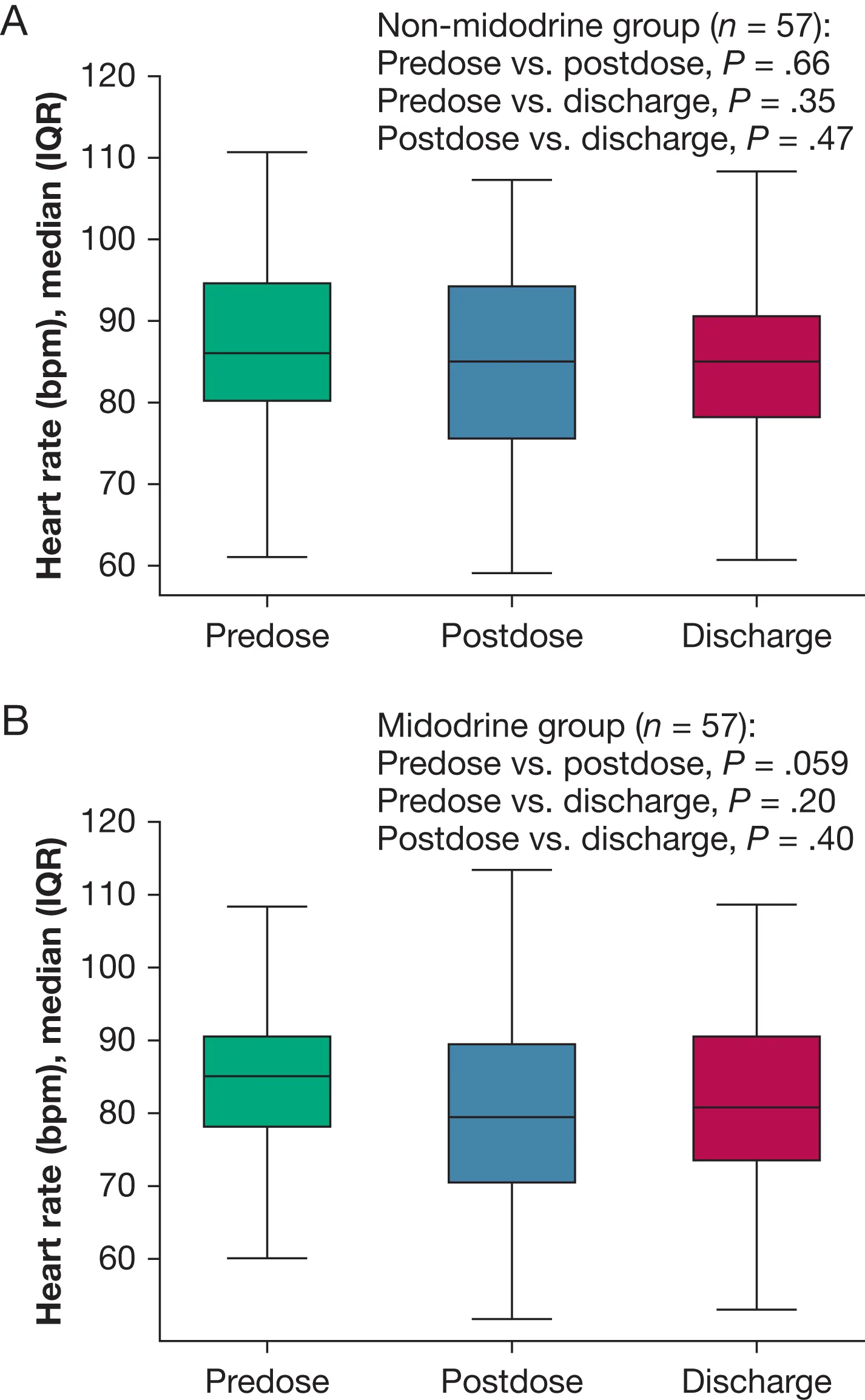
A-B, Mean heart rate at 3 time points. bpm = beats/min; IQR = interquartile range.

The MAP at different time points for both the control group and midodrine groups is shown in Figure 2 and Table 3. The average MAP on admission for the control group was 85 mm Hg (IQR, 75-90 mm Hg), which decreased to 77 mm Hg (IQR, 73-84 mm Hg) after initiating PAH therapies (*P* = .01). As expected, the midodrine group had a lower MAP before midodrine administration with a mean of 70 mm Hg (IQR, 66-75 mm Hg) compared with the control group (*P* < .001). At the predose, the average MAP was 70 mm Hg, which increased postdose to 73 mm Hg in the midodrine group (IQR, 70-79 mm Hg; *P* = .01). Compared with predose, the postdose and discharge MAP were reduced in the control group at 77 mm Hg (IQR, 73-84 mm Hg) and 78 mm Hg (IQR, 71-84 mm Hg), respectively. Furthermore, there was no difference in the discharge MAP in both the control (78 mm Hg; IQR, 71-84 mm Hg) and midodrine groups (75 mm Hg; IQR, 72-80 mm Hg; *P*= .20). The heart rate at different time points for both the control and midodrine groups is shown in Figure 3 and Table 3. Bradycardia is a known side effect of midodrine reported by Anstey et al in the Midodrine as Adjunctive Support for Treatment of Refractory Hypotension in the Intensive Care Unit: A Multicenter, Randomized, Placebo Controlled (MIDAS) trial (NCT01531959).^18^^,^^19^ The heart rate remained unchanged at predose, postdose, and discharge time points.

**Table 3:** Mean Arterial Pressure Measurements During Hospitalization

Variable	Total (n= 114)	Control Group (n = 57)	Midodrine Group (n = 57)	*P* Value
Mean arterial pressure, mm Hg				
Predose	75 (69-85)	85 (75-90)	70 (66-75)	< .001
Postdose	76 (71-82)	77 (73-84)	73 (70-79)	.01
Discharge	76 (71-81)	78 (71-84)	75 (72-80)	.20
Heart rate, beats/min				
Predose	85 (79-93)	86 (80,95)	85 (78-91)	.46
Postdose	82 (73-93)	85.0 (75-94)	79 (70-90)	.04
Discharge	82 (75-91)	85 (78-91)	81 (73-91)	.75

Data are presented as median (interquartile range). Differences between midodrine and control groups were compared by the Wilcoxon sign rank test for continuous variables.

### Discharge Medications

Changes in PAH medication during hospital stay is outlined in e-Tables 2 and 3, and Table 4. To accurately quantify dosage changes for each patient, we calculated the absolute change in dose, representing the difference between admission and discharge doses. This method accounts for new medications that are often starting at a lower dose, and could skew the average dose calculations downward, even when more patients are receiving the medication. For instance, IV epoprostenol was used equally at admission, with the control group (n = 11) averaging 22 ng/kg/min and the midodrine group (n = 9) averaging 37 ng/kg/min. There was a trend toward higher epoprostenol dosages in the midodrine group (*P* = .057). The control group experienced a dose reduction of 0.2 ng/kg/min (*P* = NS), whereas the midodrine group showed an increase in absolute mean change in dose by 7.6 ng/kg/min (*P* < .001), because 9 patients had initiation of IV epoprostenol during hospitalization, representing an average increase of 41%. For IV treprostinil, the control group’s dose remained unchanged (0.7 ng/kg/min, *P* = NS), whereas the midodrine group had an absolute mean change in dose increase of 19.3 ng/kg/min (*P* < .05), with 3 additional patients started during hospitalization.

**Table 4:** Change in PAH Medication at Discharge Compared With Admission Calculated as Absolute Difference

Medication Dose	Control Group				Midodrine Group			
	No. of Patients	Change in No. of Patients on Therapy	Absolute Mean Dose Change (% Change)	*P* Value^a^	No. of Patients	Change in No. of Patients on Therapy	Absolute Mean Dose Change (% Change)	*P* Value^a^
Prostacyclin pathway								
Epoprostenol IV, ng/kg/min	11	2	−0.2 (−1)	> .05	9	9	7.6 (41)	< .001
Treprostinil IV, ng/kg/min	9	0	0.7 (1)	NA	5	3	19.3 (34)	< .05
Treprostinil inhaled, μg/d	2	0	0 (0)	NA	5	−1	−103 (−36)	> .05^b^
Treprostinil oral, mg/d	6	1	−1.3 (−10)	> .05	8	−2	−8.2 (−49)	< .05
Selexipag oral, mg/d	8	2	280 (13)	NA	8	7	213 (17)	< .05^b^
Nitric oxide pathway								
Sildenafil, mg/d	8	1	6.6 (13)	NA	10	−3	−21.4 (−22)	> .05
Tadalafil, mg/d	21	2	2.6 (8)	> .05	9	−1	−5 (−13)	NA
Riociguat, mg/d	18	2	0.75 (14)	< .05	16	1	0.26 (5)	> .05
Endothelin pathway								
Ambrisentan, mg/d	10	2	1.25 (15)	NA	8	−1	−0.71 (−7)	NA
Macitentan, mg/d	32	5	1.35 (166)	> .05	44	2	0.4 (5)	NA

NA = Not Applicable

a Wilcoxon rank sum test was used to compare each group between admission and discharge. *P* < .05 was considered significant. The test is 2-tailed unless otherwise specified.

b One-tailed test.

Oral selexipag initiation differed between the 2 groups. In the control group, 2 patients started selexipag, resulting in an increase of the absolute mean change in dose of 280 mg/d. In the midodrine group, the number of patients using selexipag increased from 8 to 15, leading to an increase of the absolute mean change in dose of 213 mg/d (*P* < .05). For inhaled treprostinil, only a few patients in both groups were treated (2 in the control group and 5 in the midodrine group), and due to the smaller sample sizes, *P* values could not be calculated.

Oral selexipag, and IV epoprostenol and treprostinil doses were all uptitrated in the midodrine group compared with the control group (Table 4). The only medication with a decreased dose in the midodrine group was oral treprostinil, which showed a reduction in dose. The riociguat absolute mean change in dosage significantly increased in the control group. Interestingly, sildenafil was downtitrated by approximately 20 mg/d in the midodrine group. However, the average dosage for the remaining patients increased from 69 to 77 mg/d, whereas 3 patients stopped taking sildenafil, 1 patient started, and the remaining 7 either increased or maintained their doses. Finally, patients in both groups had minimal changes in ERA therapy (ambrisentan and macitentan); however, due to smaller sample sizes, *P* values could not be calculated.

Postmatching multivariable conditional logistic regression confirmed that compared with those patients who were not receiving midodrine, patients with PAH receiving midodrine were more likely to undergo uptitration of their PAH medications (OR, 2.53; 95% CI, 1.08-5.93; *P* = .03) and more likely to receive increased doses of epoprostenol (OR, 3.75; 95% CI, 1.24-11.30; *P* = .02). Patients in the midodrine group also tended to receive an increased dose of selexipag (OR, 2.50; 95% CI, 0.49-12.88; *P* = .27) or treprostinil (OR, 2.14; 95% CI, 0.67-6.77; *P* = .20); however, these differences were not statistically significant.

### Posthospitalization Hemodynamics

Invasive hemodynamics demonstrated improved cardiac output (4.5 vs 5.0 L/min) and cardiac index (2.5 vs 3.1 L/min/m^2^) compared with baseline values in the midodrine group (Table 5). Postmidodrine echocardiography parameters were similar between the control and midodrine groups (e-Table 5).

**Table 5:** Postmidodrine Hemodynamics

Variables	Total	Baseline (Midodrine)	Postmidodrine	*P* Value
	(n = 114)	(n = 57)	(n = 57)	
RA mean, mm Hg	10.0 (6.0-15.0)	8.0 (6.0-15.0)	11.0 (4.0-15.0)	.95
PA mean, mm Hg	48.0 (42.0-57.0)	49.0 (43.0-56.0)	46.5 (38.0-59.0)	.91
PCWP, mm Hg	12.0 (8.5-17.0)	12.0 (8.0-17.0)	13.0 (10.0-18.0)	.56
TD CO, L/min	4.6 (3.7-6.2)	4.5 (3.5-5.7)	5.0 (4.4-7.8)	.04
TD CI, L/min/m^2^	2.6 (2.2-3.5)	2.5 (2.0-3.2)	3.1 (2.6-3.7)	.02
HR, beats/min	81.0 (72.0-91.0)	82.0 (73.0-91.0)	80.0 (68.0-91.0)	.91
PVR, WU	7.4 (4.2-10.7)	7.4 (5.1-10.9)	6.9 (3.1-10.2)	.29
TPG, mm Hg	35.0 (25.0-44.0)	35.0 (25.0-43.0)	31.0 (24.0-47.0)	.70
DPG, mm Hg	20.0 (11.5-26.0)	20.0 (12.0-26.0)	17.0 (8.0-28.0)	.81
SV, mL	58.4 (44.9-73.6)	51.2 (39.0-67.1)	68.9 (50.3- 109.9)	.04
Pulmonary compliance, mL/mm Hg	1.1 (0.8-1.9)	1.1 (0.8-1.5)	1.5 (0.9-2.3)	.25
Arterial saturation, %	96.0 (93.0-98.0)	95.3 (93.0-98.0)	97.0 (92.0-98.0)	.76
MVO_2_, %	68.3 (59.9-73.7)	67.0 (60.3-73.7)	69.4 (59.9-71.7)	.62

Data are presented as median (interquartile range) or as otherwise indicated. Differences between midodrine and control groups were compared by the Wilcoxon sign rank test for continuous variable and by McNemar χ^2^ for categorical variables. DPG = diastolic pulmonary gradient; HR = heart rate; MVO_2_ = mixed venous oxygen saturation; PA = pulmonary artery; PCWP = pulmonary capillary edge pressure; PVR = pulmonary vascular resistance; RA = right atrium; SV = stroke volume; TD CI = cardiac index thermodilution; TD CO = thermodilution cardiac output; TPG = transpulmonary gradient; WU = Wood units.

### Mortality Data

During hospitalization, 3 patients in the control group and 5 patients in the midodrine group died (*P* = .54). At the time of last data collection, 41 of the 57 control patients with PAH and 39 of the 57 midodrine group were alive (*P* = .68).

## Discussion

To our knowledge, this study is the first comprehensive analysis of PAH data examining the use of midodrine in hospitalized patients with PAH. Our study demonstrated the safety of midodrine in hospitalized patients with PAH to improve MAP, thereby allowing uptitration of PAH therapies. Oral selexipag, and IV epoprostenol and treprostinil doses, were more likely to be uptitrated in the midodrine group compared with the control group. Oral PDE5i was the most often discontinued drug during admission. Baseline demographic data for both the control and midodrine groups showed similarities; however, differences in 6-minute walk distance, REVEAL Lite 2 score, and plasma brain natriuretic peptide levels suggested that the midodrine group had more severe disease at the time of hospitalization, requiring escalation of PAH therapy.

Common indications for midodrine use include orthostatic hypotension, liver cirrhosis, hepatorenal syndrome, and chronic heart failure.^8^, ^9^, ^10^, ^11^^,^^20^, ^21^, ^22^, ^23^, ^24^ Previously, the maximum midodrine dosage used safely without adverse effects was 30 mg/d in nondialysis patients and 25 mg/d in patients with end-stage renal disease.^10^ The average midodrine dose used in our study was 28 mg/d. One limiting factor for uptitrating PAH medications during hospitalization is a low MAP. A single case report by Drambarean et al^13^ reported the use of 60 mg/d of midodrine, with 30 mg used intradialytic for treprostinil-induced hypotension, without adverse effects. In patients with ascites and hepatorenal syndrome, midodrine has been used to improve systemic vascular resistance without compromising hemodynamics.^8^^,^^9^^,^^22^^,^^23^ Specifically, the combination of midodrine, octreotide, and albumin has been shown to improve survival and renal function in patients with hepatorenal syndrome.^9^ These results suggest that patients with hypotension may benefit from the use of midodrine therapy.

The addition of midodrine was done at the discretion of the treating physician during hospital admission. Moreover, this study was undertaken to determine the pulmonary hypertension medication dose escalation allowed by the introduction of midodrine. The time at which concern for hypotension occurred can be inferred from this dataset. Moreover, the exact criteria are difficult to elucidate. These data do suggest that the presence of clinically significant hypotension resulted in the addition of midodrine in these patients. Parental prostanoids increased with midodrine therapy, whereas PDE5i doses were likely reduced to allow uptitration of parenteral proteinoid therapy. There were only 4 patients on ERA and PDE5i therapy who were started on midodrine, and 5 patients on ERA and PDE5i therapy who were not started on midodrine. In addition, oral PDE5i was the most often discontinued drug during admission. Our data suggest that using this strategy, the PAH medications dose escalation was well tolerated. However, a well-designed prospective study would help to define cutoffs and establish criteria as to when midodrine would be most helpful in a clinical setting.

Consistent with current recommendations, most patients at baseline in this study were on double or triple PAH therapies.^25^ Of note, at the time of this analysis, sotatercept was not an approved PAH medication. There was little intergroup heterogeneity because the control and midodrine groups had a similar distribution of PAH medications at baseline. The only exception was that the control group had a higher proportion of patients who were on a nitric oxide pathway medication, namely tadalafil (n = 21 vs n = 9); however, no difference in the average daily dose was noted. In the midodrine group, prostacyclin dose increased by 17% (oral selexipag), 34% (IV treprostinil), and 41% (IV epoprostenol), and 7 new patients were initiated on selexipag and 9 new patients were initiated on IV epoprostenol. In the control group, more patients were started on oral PAH therapies (oral treprostinil, oral selexipag, riociguat, sildenafil, tadalafil, ambrisentan, and macitentan) (Table 4). This finding can be extended to hypothesize that patients treated with oral medications were more clinically stable.

**Figure 4:**
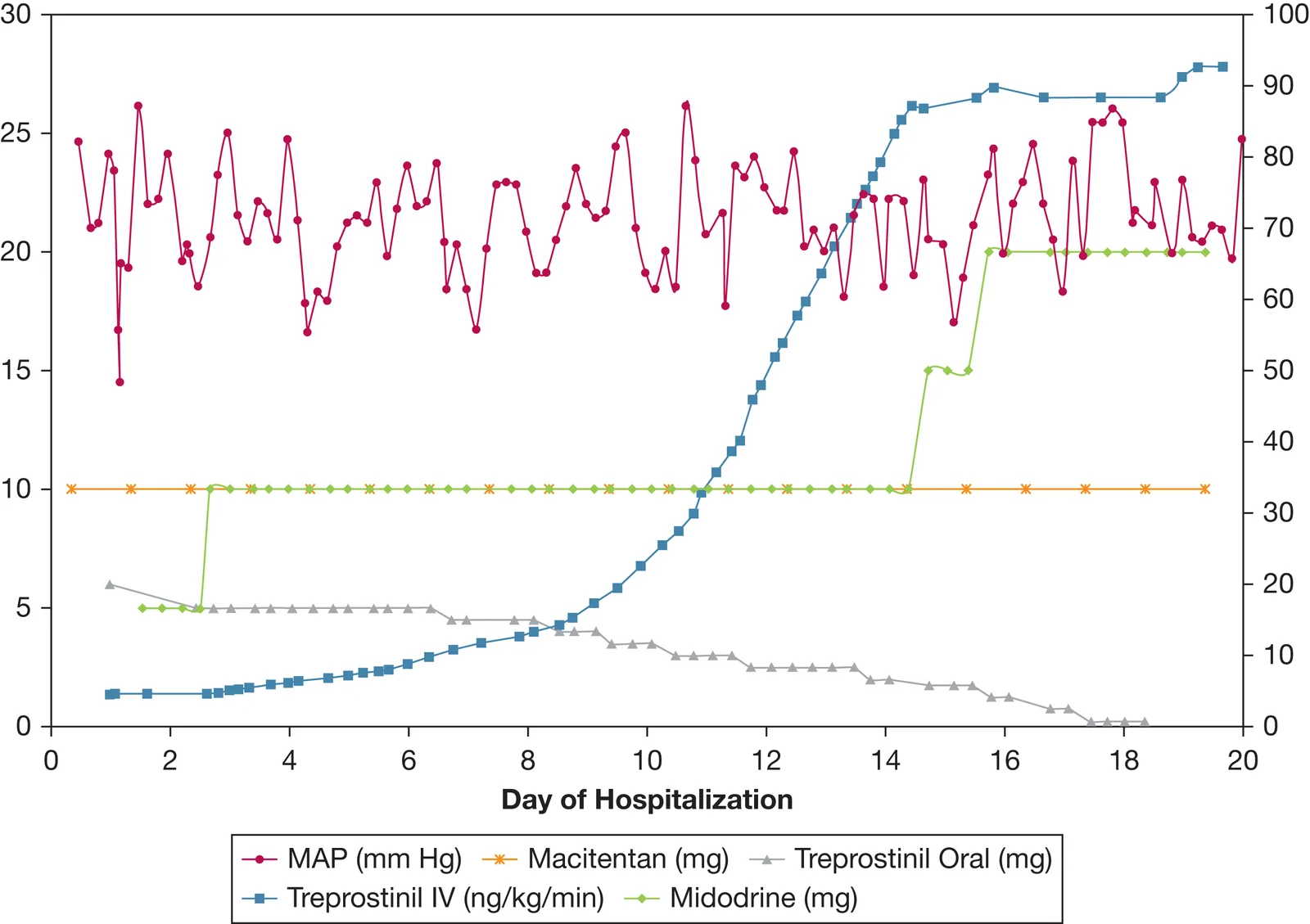
Example of patient receiving midodrine for PAH therapy titration. MAP = mean arterial pressure; PAH = pulmonary arterial hypertension.

Across both the control and midodrine groups, the number of medications and their collective dosages increased during the hospitalization and are outlined in e-Table 2 and 3. Interestingly, 1 patient in the control group and 2 in the midodrine group were switched from oral treprostinil to IV treprostinil during hospitalization. This could be due to ease of titrating IV medications compared with oral medications. With the more common use of subcutaneous treprostinil, there have been changes in median outpatient treprostinil dosages, with subcutaneous dosages titrated to higher doses than IV treprostinil.^26^ For hospitalized patients with PAH experiencing side effects, switching to IV treprostinil may be a strategy to rapidly uptitrate this regimen. Figure 4 depicts a patient who was admitted and underwent cross titration from oral treprostinil to IV treprostinil with the assistance of midodrine to avoid hypotension. This study did not examine how often IV treprostinil was weaned back to oral treprostinil on discharge.

One limitation of the study was that the midodrine group was self-selecting, in that only patients with lower MAP with or without symptoms of hypotension were started on midodrine. There was no difference in mortality between patients in the control and midodrine groups. Of note, most patients who present to the hospital have multiple hospitalization events, and to reduce randomness and investigator bias, the most recent hospitalization event was used for this analysis. Another important limitation was in some instances the sample size of a medication is < 10, which may result in type 1 or type 2 errors. Common side effects of midodrine include bradycardia, hypertension, urinary frequency, and retention.^24^ By promoting vasoconstriction and venoconstriction, midodrine can effectively increase systemic vascular resistance and MAP.^8^^,^^9^^,^^14^^,^^15^^,^^22^^,^^27^ Finally, no side effects were reported during the hospitalizations with the administration of midodrine. Side effects that were attributed to the titration of PAH therapies (based on available documentation) were not reported. In this descriptive retrospective study, it is hard to determine the exact time period at which midodrine was started, and the exact reasoning behind starting midodrine in the inpatient setting. It is likely that patients with symptomatic hypotension triggered the treating physician to add midodrine to allow uptitration of pulmonary hypertension medications. Our data suggest that the dose escalation in the midodrine group was well tolerated. Due to the nature of this study, any demonstrated benefit in mortality, change in hemodynamics, or clinical status should be attributed to the PAH therapy themselves. The addition of midodrine would not be responsible for any clinical improvement, rather it is the optimization of maximally tolerated PAH therapy. Moreover, the long-term effect of midodrine on the disease and mortality needs further investigation. In this study, we applied propensity score matching from the outset as part of the study design. Given that this method is well established and widely accepted for balancing covariates in observational studies, our analysis was focused within this framework. Exploring alternative matching methods would require restructuring the analytical approach and is beyond the scope of this study. Future work could explore the robustness of findings using different matching strategies. In addition, mixed-effects modeling is beyond the scope of the current study because the analysis focuses on comparisons between baseline and postmidodrine time points. Additionally, the lack of repeated measures in many patients supports our chosen approach. This is a potential avenue for future studies with more complex longitudinal data structures.

Hemodynamic data from both echocardiogram and right heart catheterization is often used to direct medical therapy, assess effectiveness, and in combination with scoring tools help to assess treatment response to PAH therapy. Cardiac output and cardiac index improvement noted in the midodrine group likely represent a response to PAH therapy escalation. This study demonstrates that midodrine did not have a deleterious effect on the PAH cohort. However, an individualized treatment approach is required in treatment decisions.

## Interpretation

This study demonstrates that midodrine was efficacious at improving MAP, thereby allowing uptitration of PAH medication, and this effect was maintained until the time of discharge. Furthermore, midodrine was well tolerated and safe, with no adverse effects reported. Although not examined in this study, it is reasonable to continue using midodrine after discharge. Further studies are needed to determine the long-term effect of midodrine in patients with PAH. A well-designed prospective study would help to define the use of midodrine in patients with PAH.

## Funding/Support

The authors have reported to *CHEST Pulmonary* that no funding was received for this study.

## Financial/Nonfinancial Disclosures

The authors have reported to *CHEST Pulmonary* the following: Z. S. reports a relationship with Boehringer Ingelheim Pharmaceuticals Inc, Genetech Biotech Co Ltd, and Actelion Pharmaceuticals US Inc that includes consulting or advisory and speaking and lecture fees; reports a relationship with United Therapeutics Corp and HealthCare Pharmaceuticals Inc that includes consulting or advisory; and reports a relationship with Merck Pharma and Chemicals that includes speaking and lecture fees. None declared (N. G., A. M., S. M., N. V. H., D. T. N., E. N. G.).

## Supplementary Data

e-Online Data

e-Online Data

e-Online Data

e-Online Data

e-Online Data
