# Supplementary material for: Real-World Experience of Midodrine in Hospital Setting in Pulmonary Arterial Hypertension
Source: CHEST Pulm. 2025 Sep 30;4(3):100214. doi: 10.1016/j.chpulm.2025.100214 (PMC13534841; doi:10.1016/j.chpulm.2025.100214)
Supplement: Supplementary file 1 [file mmc1.docx]

Supplement Table 1. Baseline Echocardiogram

|  | Total | Control | Midodrine | *P*-value |
| --- | --- | --- | --- | --- |
|  | N=114 | N=57 | N=57 |  |
| RA Area (cm^2^) (n=68) | 24.6 (19.8, 31.9) | 22.0 (16.0, 29.0) | 25.8 (22.5, 32.8) | 0.04 |
| RA index (cm^2^/m^2^) (n=65) | 49.0 (34.0, 70.0) | 45.0 (30.1, 52.1) | 57.0 (43.0, 79.0) | 0.04 |
| RA volume (mL) (n=67) | 89.0 (55, 134) | 80.8 (44, 106) | 100.0 (76, 142) | 0.03 |
| PASP (mmHg) (n=74) | 70.5 (57.0, 93.0) | 70.0 (63.0, 82.0) | 73.0 (56.5, 94.5) | 0.82 |
| Stroke volume index (ml/m^2^) | 33.0 (28.0, 40.0) | 36.9 (32.0, 42.0) | 32.0 (26.6, 36.0) | 0.03 |
| Pericardial Effusion (n, %) |  |  |  | 0.47 |
| No | 63 (64) | 30 (68) | 33 (61) |  |
| Yes | 35 (36) | 14 (32) | 21 (39) |  |
| TAPSE mm (n=56) | 1.9 (1.4, 2.2) | 2.0 (1.5, 2.3) | 1.6 (1.2, 2.1) | 0.05 |
| LV ejection fraction (%) | 69.0 (64.0, 70.0) | 65.0 (64.0, 69.0) | 69.0 (64.0, 70.0) | 0.22 |
| LA Area (cm^2^) (n=74) | 18.0 (15.0, 22.0) | 17.3 (15.0, 20.2) | 19.0 (15.0, 26.4) | 0.15 |
| LVOT stroke volume (ml) (n=75) | 56.4 (47.0, 75.0) | 60.0 (51.0, 76.2) | 53.9 (46.4, 70.0) | 0.49 |
| LVOT cardiac output (L/min) (n=77) | 4.3 (3.6, 5.2) | 4.5 (3.8, 5.2) | 4.3 (3.3, 5.6) | 0.48 |
| LVOT cardiac index (L/min/m^2^) (n=75) | 2.6 (2.3, 3.2) | 2.5 (2.2, 3.2) | 2.7 (2.3, 3.2) | 0.99 |
| Heart rate bpm (n=77) | 78.0 (67.0, 92.0) | 78.0 (66.0, 84.0) | 79.0 (70.0, 99.0) | 0.31 |

*****Values are reported as median and interquartile range (IQR) for continuous variables and as frequencies and proportions for categorical variables unless otherwise specified; Differences between midodrine and control groups were compared by the Wilcoxon sign-rank test for continuous variable and by McNemar's chi-squared for categorical variables. LA, left atrial; LV, left ventricle; LVOT, left ventricular outflow tract; PASP, pulmonary artery systolic pressure; RA=right atrium; RV S', right ventricular systolic velocity; TAPSE, tricuspid annular plane systolic
