# Supplementary material for: Real-World Experience of Midodrine in Hospital Setting in Pulmonary Arterial Hypertension
Source: CHEST Pulm. 2025 Sep 30;4(3):100214. doi: 10.1016/j.chpulm.2025.100214 (PMC13534841; doi:10.1016/j.chpulm.2025.100214)
Supplement: Supplementary file 2 [file mmc2.docx]

e-Table 2. Post-midodrine Echocardiogram

|  | Total | Baseline | Post-Midodrine | *P-*value |
| --- | --- | --- | --- | --- |
|  | N=114 | N=57 | N=57 |  |
| RA Area (cm^2^) | 25.8 (21.0, 32.0) | 25.8 (22.5, 32.8) | 25.8 (20.0, 30.0) | 0.38 |
| RA volume (mL) | 92.8 (66.8, 134.0) | 100.0 (76.1, 142.0) | 89.8 (62.0, 121.0) | 0.26 |
| RA index (cm^2^/m^2^) | 56.0 (38.5, 70.0) | 57.0 (43.0, 79.0) | 49.9 (35.9, 69.0) | 0.32 |
| PASP (mmHg) | 90.0 (75.5, 103.0) | 89.2 (59.7, 105.0) | 90.0 (90.0, 90.0) | 1.00 |
| Pericardial Effusion (n, %) |  |  |  | 0.74 |
| No | 59 (60) | 33 (60) | 26 (58) |  |
| Yes | 40 (40) | 21 (40) | 19 (42) |  |
| TAPSE mm (n=56) | 1.9 (1.3, 2.2) | 1.6 (1.2, 2.1) | 1.9 (1.4, 2.3) | 0.27 |
| RV S’ cm/s(n=21) | 9.9 (7.9, 12.0) | 9.7 (7.9, 12.0) | 9.9 (7.9, 12.2) | 0.92 |
| LV ejection fraction (%) | 69.0 (64.0, 70.0) | 69.0 (64.0, 70.0) | 69.0 (64.0, 70.0) | 0.86 |
| LA Area (cm^2^ ) | 18.0 (15.0, 23.1) | 19.0 (15.0, 26.4) | 16.9 (14.0, 21.9) | 0.15 |
| LVOT stroke volume (mL) | 54.1 (45.2, 72.0) | 53.9 (46.4, 70.0) | 54.1 (42.0, 72.0) | 0.73 |
| LVOT cardiac output (L/min) | 4350.0 (3300.0, 5400.0) | 4300.0 (3300.0, 5600.0) | 4500.0 (3100.0, 5400.0) | 0.88 |
| LVOT cardiac index (L/min/m^2^) | 2.6 (2.2, 3.2) | 2.7 (2.3, 3.2) | 2.6 (2.1, 3.2) | 0.76 |
| Stroke volume index (ml/m^2^) | 32.6 (26.0, 37.0) | 32.0 (26.6, 36.0) | 33.3 (26.0, 40.0) | 0.78 |
| Heart rate (bpm) | 81.0 (67.5, 96.5) | 79.0 (70.0, 99.0) | 82.0 (67.0, 96.0) | 0.88 |

*****Values are reported as median and interquartile range (IQR) for continuous variables and as frequencies and proportions for categorical variables unless otherwise specified; Differences between midodrine and control groups were compared by the Wilcoxon sign-rank test for continuous variable and by McNemar's chi-squared for categorical variables. LA, left atrial; LV, left ventricle; LVOT, left ventricular outflow tract; PASP, pulmonary artery systolic pressure; RA=right atrium; RV S', right ventricular systolic velocity; TAPSE, tricuspid annular plane systolic
