# Supplementary material for: Real-World Experience of Midodrine in Hospital Setting in Pulmonary Arterial Hypertension
Source: CHEST Pulm. 2025 Sep 30;4(3):100214. doi: 10.1016/j.chpulm.2025.100214 (PMC13534841; doi:10.1016/j.chpulm.2025.100214)
Supplement: Supplementary file 3 [file mmc3.docx]

**e-Table 3.** Distribution of medication regimen at discharge; non-midodrine vs. midodrine

|  | Total  N=114 (%) | Control  N=57 (%) | Midodrine  N=57 (%) | *P*-value |
| --- | --- | --- | --- | --- |
| Medications |  |  |  | 0.07 |
| PCA | 96 (84) | 44 (77) | 52 (91) | 0.04 |
| ERA | 102 (89) | 49 (86) | 53 (93) | 0.22 |
| sGC | 37 (32) | 20 (35) | 17 (30) | 0.55 |
| PDE5i | 47 (41) | 32 (56) | 15 (26) | 0.001 |
| Number of medications (PCA, ERA, or sGC/PDE5i) |  |  |  | 0.23 |
| 1 | 4 (4) | 2 (4%) | 2 (4) | 0.65 |
| 2 | 56 (49) | 24 (42) | 33 (58) | 0.13 |
| 3 | 53 (46) | 31 (54) | 22 (39) | 0.09 |
| Treatment specified |  |  |  |  |
| PDE5i only | 1 (1) | 1 (2) | 0 | -- |
| ERA only | 2 (2) | 0 | 1 (2) | -- |
| PCA only | 2 (2) | 1 (2) | 1 (2) | 1.00 |
| PDE5i + ERA | 13 (11) | 9 (16) | 4 (7) | 0.14 |
| PDE5i + PCA | 7 (6) | 5 (9) | 2 (4) | 0.24 |
| ERA + PCA | 26 (23) | 4 (7) | 23 (40) | <0.0001 |
| ERA + sGC | 8 (7) | 5 (9) | 3 (5) | 0.46 |
| PCA + sGC | 2 (2) | 1 (2) | 1 (2) | 1.00 |
| PDE5i + ERA + PCA | 26 (23) | 17 (30) | 9 (16) | 0.07 |
| ERA + PCA + sGC | 27 (24) | 14 (25) | 13 (23) | 0.83 |

* Values are in number (%) unless otherwise specified; IQR, interquartile range; PCA, prostacyclin analog; ERA, endothelin-receptor antagonist; sGC, soluble guanylyl cyclase; PDE5i, phosphodiesterase-5 inhibitor. Differences between midodrine and control were compared by chi-squared test of independence.
