# Supplementary material for: Real-World Experience of Midodrine in Hospital Setting in Pulmonary Arterial Hypertension
Source: CHEST Pulm. 2025 Sep 30;4(3):100214. doi: 10.1016/j.chpulm.2025.100214 (PMC13534841; doi:10.1016/j.chpulm.2025.100214)
Supplement: Supplementary file 4 [file mmc4.docx]

**e-Table 4**. Characterization of PAH therapies while hospitalized

|  | Started | Up Titrate | Stable | Down Titrate | Dis-continued | Changed* | None |  |
| --- | --- | --- | --- | --- | --- | --- | --- | --- |
| Control  – Individual medications |  |  |  |  |  |  |  |  |
| PCA | 5 | 5 | 24 | 1 | 0 | 7 | 15 |  |
| ERA | 10 | 0 | 39 | 0 | 2 | 0 | 6 |  |
| PDE5i | 3 | 2 | 25 | 2 | 0 | 0 | 25 |  |
| sGC | 3 | 3 | 14 | 0 | 0 |  | 37 |  |
|  | Up Titrate | | Stable | Down Titrate | | Changed* |  |  |
| Overall Hospitalization Experience | 17 | | 29 | 3 | | 8 |  |  |
|  | | | | | | | | |
| Midodrine - Individual medications |  |  |  |  |  |  |  | *P*-value |
| PCA | 7 | 19 | 10 | 1 | 1 | 11 | 8 | <.001 |
| ERA | 3 | 1 | 48 | 0 | 1 | 1 | 3 | 0.17 |
| PDE5i | 1 | 2 | 10 | 2 | 5 | 0 | 37 | 0.01 |
| sGC | 3 | 3 | 11 | 0 | 3 |  | 37 | 0.99 |
| Overall Hospitalization Experience | 30 | | 15 | 3 | | 9 |  | 0.043 |

Values are in number unless otherwise specified. PCA, prostacyclin analog; ERA, endothelin-receptor antagonist; sGC, soluble guanylyl cyclase; PDE5i, phosphodiesterase-5 inhibitor. Differences between midodrine and control were compared by chi-squared test goodness of fit. Categories that contain a 0 were excluded. *A patient was categorized as "changing" their PH medication regimen when they switched from one drug to another within the same class, and by the time of discharge were on equivalent dosages.
