# Supplementary material for: Real-World Experience of Midodrine in Hospital Setting in Pulmonary Arterial Hypertension
Source: CHEST Pulm. 2025 Sep 30;4(3):100214. doi: 10.1016/j.chpulm.2025.100214 (PMC13534841; doi:10.1016/j.chpulm.2025.100214)
Supplement: Supplementary file 5 [file mmc5.docx]

**e-Table 5. PAH medication doses on admission**

| Medication Dose | On Admission | | | | |
| --- | --- | --- | --- | --- | --- |
|  | Control Group | | Midodrine Group | | *P*-value |
|  | Number (n) | Average Dose | Number (n) | Average Dose |  |
| Prostacyclin Pathway |  |  |  |  |  |
| Epoprostenol IV (ng/kg/min) | 11 | 22 | 9 | 37 | 0.06 |
| Treprostinil IV (ng/kg/min) | 9 | 69 | 5 | 92 | 0.42 |
| Treprostinil Inhaled (mcg/day | 2 | 168 | 5 | 230 | -- |
| Treprostinil Oral (mg/day) | 6 | 16 | 8 | 13 | 0.47 |
| Selexipag oral (mg/day) | 8 | 2700 | 8 | 2350 | 0.56 |
| Nitric Oxide Pathway |  |  |  |  |  |
| Sildenafil (mg/day) | 8 | 58 | 10 | 69 | 0.69 |
| Tadalafil (mg/day) | 21 | 38 | 9 | 36 | 0.60 |
| Riociguat (mg/day) | 18 | 6 | 16 | 6 | 0.56 |
| Endothelin Pathway |  |  |  |  |  |
| Ambrisentan (mg/day) | 10 | 10 | 8 | 10 | 0.68 |
| Macitentan (mg/day) | 32 | 10 | 44 | 10 | 0.99 |

*Mann Whitney U test was used to compare each group at admission. P value <0.05 was considered significant.
